# Endoscopic resection through a retrosigmoid transmeatal approach of a large-sized vestibular schwannoma: operative video and technical nuances

**DOI:** 10.3171/2021.7.FOCVID21106

**Published:** 2021-10-01

**Authors:** Sebastián J. M. Giovannini, Guido Caffaratti, Tomas Ries Centeno, Mauro Ruella, Facundo Villamil, Ricardo Marengo, Andrés Cervio

**Affiliations:** Department of Neurological Surgery, FLENI, Buenos Aires, Argentina

**Keywords:** vestibular schwannoma, endoscopic approach, retrosigmoid approach, facial nerve

## Abstract

Surgical management of vestibular schwannomas has improved over the last 30 years. Whereas in the past the primary goal was to preserve the patient’s life, today neurological function safeguarding is the main objective, with numerous strategies involving single resection, staged resections, postoperative radiosurgery, or single radiosurgery.

The retrosigmoid approach remains the primary pathway for surgical access to the cerebellopontine angle (CPA). The use of an endoscope has great advantages. It contributes to the visualization and resection of residual tumor and also reduces the need for cerebellar retraction.

The authors present a fully endoscopic resection of a large-sized vestibular schwannoma with facial nerve preservation.

The video can be found here: https://stream.cadmore.media/r10.3171/2021.7.FOCVID21106

## Transcript

In this video, we present a fully endoscopic resection of a large-sized vestibular schwannoma through a retrosigmoid transmeatal approach.

### 0:30 Clinical Presentation.

This is a case of a 47-year-old female patient who presented with tinnitus and progressive hearing loss on the right side. Preoperative audiogram revealed a grade C of the AAO-HNS hearing classification.

### 0:44 Preoperative Imaging.

Preoperative MRI showed a right T3b vestibular schwannoma protruding from the IAC into the CPA.

In this case, we decided to perform a right-sided retrosigmoid trasmeatal approach based on the location and extension of the tumor.^1–3^ We believe that the use of the endoscope reduces the need for cerebellar retraction and excessive bone drilling.^3–5^

### 1:08 Positioning and Opening Technique.

The patient is placed in the park-bench position and all pressure points are carefully padded. For facial nerve monitoring, bipolar electrodes are placed in facial muscles.

A retroauricular C-shaped incision is made, and a musculocutaneous flap is elevated to expose the suboccipital bone. Bony landmarks are identified and a right suboccipital retrosigmoid craniectomy is then performed.

Draining of CSF from the lateral cerebellomedullary cistern allows the cerebellum to fall away from the petrous bone, facilitating the dural opening, which is then performed along the sigmoid and transverse sinuses to create a posteriorly based C-shaped dural flap. Note that the cerebellar relaxation creates a wider corridor for the introduction of the endoscope, avoiding cerebellar retraction.

We implemented the freehand dynamic endoscopic technique, which consists in the cooperation of an assistant which holds and directs the endoscope while the main surgeon performs the dissection with two hands.

### 2:10 Identification of Tumor Within the CPA.

A 0° endoscope is carefully placed in the cerebellopontine angle and the tumor is identified below the suprameatal tubercle.

The petrosal vein is visualized superiorly and the lower cranial nerves inferiorly, coursing toward the jugular foramen. The posterior surface of the tumor is stimulated to exclude a posterior displaced facial nerve. Then the tumor is coagulated with the bipolar forceps and its capsule is sharply fenestrated in a region with negative response to stimulus. Biopsy is taken for analysis.

### 2:44 Tumor Debulking.

Afterward, the tumor is internally debulked using an ultrasonic aspiration, making an effort to stay within the tumor capsule to avoid damage to cranial nerves potentially splayed over the tumor.

Using a microforceps, the arachnoid attachments are gentle dissected. Further stimulation is used to identify the facial nerve proximally, near its exit from the brainstem. The endoscope provides a wide field of vision that results very useful for identifying structures.

The tumor is gently dissected from the brainstem by following the arachnoid plane. Care is taken to preserve the arachnoid overlying the facial nerve. Further debulking allows mobilization and blunt dissection of the tumor. After sufficient debulking, the IAC must be drilled.

### 3:35 Drilling of the IAC.

The posterior wall of the acoustic canal is identified and its dura is coagulated and cut. Afterward, the opening of the internal auditory canal is performed with the use of a high-speed drill with constant irrigation. During the drilling we find useful to put the retractor only to protect the cerebellum from the drill handpiece. Initially, bone cutting and later diamond tips are used in order to achieve diligent drilling. Bone is removed 180° to 270° around the nerves. A smaller diamond burr is used to expose the very lateral aspect of the IAC and its contents.

The tumor is now well exposed and gently dissected away from the canal with preservation of the facial nerve. Stimulation is used to confirm its location. The tumor is further dissected and delivered away from the fundus. A hook dissector is used to certify the absence of residual tumor in the IAC. The cochlear nerve is observed at its normal caudal position.

### 4:43 Final Tumor Resection.

After the intracanalicular tumor has been removed, circumferential dissection continues in the CPA. Sharp meticulous dissection from lateral to medial using the plane between the arachnoid layers and tumor debulking is very helpful at this time. When having concerns about the location of the facial nerve, a higher-intensity setting during stimulation to check the potential course of the seventh nerve can be useful.

The facial nerve can be strongly tethered to the tumor in the porus. In such cases, we prefer to continue dissecting from medial to lateral, starting where the nerve emerges from the brainstem toward the IAC. Then the final attachment of the tumor can be coagulated and cut. Blunt dissection with microsurgical instruments progresses in this fashion until the two dissection planes meet. Then the final debulking is performed, leaving a minimum remnant in contact with the facial nerve.

Stimulation of the facial nerve with 0.3 mA or less is a good predictor of preserved postoperative facial function.

We can finally check with the endoscope the surgical lodge. Hemostasis is achieved with Surgicel; bipolar cautery should be avoided around the facial nerve.

With the improved vision provided by the endoscope, we confirm both the presence of air cells in the drilled IAC and the absence of residual tumor in the fundus.

In cases with large air cells in order to reduce the risk of CSF leak, bone wax is often used as well as a muscle or fat graft secured with fibrin glue. In this case, we didn’t find it necessary and only Surgicel was placed.

### 6:32 Closure.

Dura is finally closed in a watertight fashion, and bone cement cranioplasty is then placed at the craniectomy site. Standard muscle and skin closure is performed.

### 6:42 Postoperative Course and Postoperative Imaging.

Postoperative MRI showed the remotion of the lesion without any complication.

The patient evolved favorably, with a preserved facial function. Postoperative hearing progressed to right cophosis. She was discharged on day 4.

In this video, we can see the technical nuances of a fully endoscopic-assisted resection of large-sized vestibular schwannoma with facial nerve preservation.

## Disclosures

The authors report no conflict of interest concerning the materials or methods used in this study or the findings specified in this publication.

## Author Contributions

Primary surgeon: Cervio. Assistant surgeon: Giovannini, Ruella, Marengo. Editing and drafting the video and abstract: Giovannini, Caffaratti, Ries Centeno, Ruella, Villamil. Critically revising the work: Giovannini, Caffaratti, Ries Centeno, Ruella, Villamil, Cervio. Reviewed submitted version of the work: Caffaratti, Ries Centeno, Ruella, Villamil. Approved the final version of the work on behalf of all authors: Giovannini.
